# Establishment of an anaplastic stratification signature for gastric cancer based on diverse regulated cell-death

**DOI:** 10.3389/fimmu.2025.1606789

**Published:** 2025-09-10

**Authors:** Shaofei Chen, Zhiyong Wang

**Affiliations:** Department of Gastrointestinal Surgery, Union Hospital, Tongji Medical College, Huazhong University of Science and Technology, Wuhan, China

**Keywords:** gastric cancer, regulated cell death, prognosis, immunotherapy efficacy, TRIML2

## Abstract

**Background:**

Gastric cancer is a common malignant tumor characterized by poor prognosis and limited therapeutic options. The combination of Regulated cell death inducers and enhancement of the immune therapeutic effect plays an important role in cancer treatment.

**Methods:**

We downloaded and analyzed data from gastric cancer samples, collected 14 Regulated cell death-related genes and constructed a Regulated Cell Death-Related Index (RCDRI) by various machine learning methods. Based on the RCDRI, gastric cancer patients were divided into high RCDRI and low RCDRI groups, and the clinical characteristics, immune cell infiltration, chemotherapy response and immunotherapy response of gastric cancer patients were analyzed based on the RCDRI.

**Results:**

The newly constructed RCDRI consisted of four Regulated cell death-related genes (CD36, SERPINE1, TRIML2, and GRP) and has been shown to be an effective predictive marker for the survival of gastric cancer patients and was trained with multiple external datasets. The high RCDRI group had a higher level of immune cell infiltration and better response to immunotherapy than the low RCDRI group. In addition, through pan-cancer analysis, we found that RCDRI can also be used for prognosis and immunotherapy prediction in a variety of cancers. Finally, *in vitro* experiments revealed that TRIML2 knockdown inhibited the proliferation and migration of gastric cancer cells.

**Conclusions:**

The RCDRI identified in this study can accurately assess the prognosis and immunotherapy efficacy of gastric cancer patients, which lays a valuable foundation for future clinical treatment of gastric cancer.

## Introduction

Gastric cancer (GC) is a prevalent kind of solid tumors that is widespread around the globe (1). Individuals with advanced stages of the illness continue to have a considerable risk of recurrence and death. This is mostly because to the extensive diversity seen among GC cells. Given the circumstances, it is crucial to promptly discover first biomarkers and choose the therapeutic approach based on each patient’s specific condition.

Cell death may occur via two primary mechanisms: accidental cell death, which is an uncontrolled biological event, and regulated cell death (RCD), which is a sophisticated death program. RCD, or regulated cell death, encompasses several cellular processes (2). Apoptosis is a natural, deliberate process of cell self-destruction. Under certain internal and external circumstances, the process of cell death is triggered without inducing inflammation (3). Pyroptosis is a kind of controlled cell death that relies on caspase-1 and results in the production of several pro-inflammatory substances, particularly in response to microbial infections (4). Autophagy is a controlled process of cell death where defective proteins or organelles are enclosed by double-membrane autophagic vacuoles for the purpose of breakdown and recycling (5). Lysosome-dependent cell death is facilitated by hydrolases that are stored in lysosomes and transported to the cytoplasm upon membrane permeabilization (6). Necroptosis is a caspase-independent process that leads to the rupture of the cell membrane, which is a specific morphological characteristic that may trigger inflammation (5). Ferroptosis is a kind of controlled cell death that relies on iron and leads to excessive lipid peroxidation and consequent cell dysfunction (7). Copper death refers to a regulatory process of cell death that occurs due to an excessive buildup of copper. This phenomenon is strongly associated with several disorders (8). Entotic cell death result in the destruction of the intracellular cell structure (9). Parthanatos is a kind of regulated cell death (RCD) that occurs as a result of DNA damage and the activation of PARP-1 (10). Netotic cell death is triggered by the liberation of neutrophil extracellular traps (NETs) (2). Alkaliptosis is a newly discovered kind of regulated cell death (RCD) that is triggered by an increase in intracellular pH (alkalinization) (11). Oxeiptosis employs reactive oxygen species (ROS) to coordinate the cellular death process (12). Apoptosis is a significant process that protects against cancer. Nevertheless, there have been few investigations on the effectiveness of tumor immunotherapy via the regulation of cell death (13). While the majority of regulated cell death routes in GC have been well investigated, the precise involvement of regulated cell death integration in GC remains uncertain (14). Thus, it is important to assess the prognosis and therapy of GC patients by examining the genes associated with controlled cell death pathways.

This work included gathering genes associated with various regulated cell death pathways and using several machine learning techniques to create a novel metric, known as the regulated cell death-related index (RCDRI). The purpose of this index was to predict the prognosis and assess the efficacy of therapy for patients with gastric cancer. Ultimately, *in vitro* investigations were used to confirm the functional significance of TRIML2 in the advancement of gastric cancer.

## Materials and methods

### Acquire genomic data from patients diagnosed with stomach cancer

The major regulatory genes of 14 regulated cell death patterns were compiled from the GSEA gene collection and several published studies as genes associated to regulated cell death. The ultimate gene list consists of a series of 14 regulatory genes with specific RCD patterns (2, 8–14). These patterns are detailed in **Supplementary Table S1**. Furthermore, the accuracy of the training set was confirmed by obtaining pertinent data from the TCGA-STAD(N = 32, T = 375) (15) for patients with stomach cancer and from the GEO database (ID: GSE84437, N = 433) for gastric cancer as a validation group. General characteristics of the two cohorts are outlined in **Supplementary Table S2**.

### Development of the regulated cell death-related index

We utilized differential analysis between gastric cancer tissue and normal tissue, setting the threshold for differential logFC greater than 2 and p adj less than 0.01. Additionally, univariate Cox regression was used to assess the association between RCD-related genes and survival status in gastric cancer patients, with the cutoff p-value adjusted to 0.05. Using the R package “glmnet,” these DEGs were subjected to a minimum absolute shrinkage and selection operator (LASSO) penalized Cox regression analysis to construct the most appropriate signature by narrowing down the range of candidate genes. The normalized expression levels of candidate DEGs and survival data (time and status) served as the independent and dependent variables, respectively, in the LASSO regression. The penalty parameter (λ) was determined using 10-fold cross-validation with the lowest standard. A risk score was calculated for each patient based on the expression levels of the DEGs and their corresponding coefficients. The Regulated Cell Death-Related Index (RCDRI) for each patient is ultimately obtained using the following formula: Regulated Cell Death-Related Index (RCDRI) = Coef(Gene 1) × Expr(Gene 1) + Coef(Gene 2) × Expr(Gene 2) +… + Coef(Gene n) × Expr(Gene n). Where Coef(Gene) represents the risk regression coefficient of Gene, and Expr(Gene) represents the expression level of Gene. Additionally, principal component analysis (PCA) and t-distributed stochastic neighbor embedding (t-SNE) were performed to further visualize the spatial dimensions between risk groups. PCA was performed using the “prcomp” function in the ‘stats’ R package, and t-SNE was performed using the “Rtsne” package in R. All steps were repeated in the GEO cohort for validation. Gastric cancer patients(TCGA-STAD)were divided into two groups based on the median RCDRI value: the high RCDRI group and the low RCDRI group.

### Analysis of the immune microenvironment

Using the expression profile as input, call the estimate R package to score stromal cells (StromalScore), immune cells (ImmuneScore), comprehensive score (ESTIMATEScore, the sum of stromal cell score and immune cell score), and tumor purity (TumorPurity) (16). The CIBERSORT method was used to measure the ratio of various immune invading cells. The ssGSEA method was used to measure the relative abundance of immune cells and immunological-related processes. The IMvigor210 study cohort evaluated the efficacy of atezolizumab (a PD-L1 targeting antibody) in 210 patients with locally advanced or metastatic uroepithelial cancer. The Kim study cohort of patients with metastatic gastric cancer treated with pembrolizumab (an anti-PD-1 inhibitor). In addition, we collected genes that have been currently reported to be positively associated with immuno-efficacy and negatively associated with immuno-efficacy and analyzed them in association with RCDRI.

### Pharmaceutical responsiveness and immune-based treatment

The response of gastric cancer patients (TCGA-STAD, n=375) to common chemotherapeutic agents was evaluated by calculating IC50 values using the R package “pRRophetic” to assess drug sensitivity to chemotherapy in gastric cancer patients with high/low RCDRI.

The calculation of the TIDE score is mainly based on the Tumor Immune Dysfunction and Exclusion (TIDE) algorithm, which first obtains the gene expression data of the tumor samples, usually RNA sequencing data and so on. The data are cleaned and normalized, usually by mean normalization, i.e., the mean value is calculated by rows, and then the mean value is subtracted from the expression of each sample. The CTL (Cytotoxic T Lymphocytes) levels of the patients were assessed using the average expression levels of the cell type markers (CD8A, CD8B) for CD8+ T cells versus the cytotoxic function markers (GZMA, GZMB, PRF1). Patients were categorized into two groups of high and low CTL according to the median CTL level. The Pearson correlation between the expression profile and T cell inactivation signature for each patient was used as the TIDE score for patients in the high CTL group. TIDE and IPS quantitation data may be used to deduce the patient’s response to immunotherapy, namely anti-PD-1 and anti-CTLA4 immunotherapy (17, 18).

### Preparation of cell lines and lentivirus infection

The cell lines MKN45 and HGC27 used in this experiment were acquired from ATCC. The aforementioned cell lines have been authenticated and preserved in the laboratory’s liquid nitrogen tank. GeneChem, located in Shanghai, China, manufactured the negative (shNC) and lentivirus-delivered shRNAs targeting TRIML2 (shTRIML2). The target sequences for shTRIML2 may be found in **Supplementary Table S3**.

### qRT-PCR

The RNA was isolated from the cells using the TRIzol technique, and the concentration and purity of the RNA were assessed. Following the conversion of RNA to complementary DNA (cDNA), quantitative polymerase chain reaction (PCR) was conducted. The primer sequences used in the study are shown in **Supplementary Table S3**.

### Experiments on cellular functions

This research conducted a CCK8 test, EdU assay, cell scratch assay, and Transwell migration invasion assay. The CCK8 experiment included the addition of MKN45 and HGC27 cells to a 96-well plate. Following a 4-hour period of cell attachment, CCK8 was introduced. The cells were then incubated at a temperature of 37°C for 1 hour, after which the absorbance was measured using a microplate reader. An experiment was conducted using logarithmic growth group cells. EdU was introduced into the culture media to facilitate EdU labeling. Following the washing of the cells with PBS, the cells were subsequently fixed, stained with fluorescent, and then photographed. Cell scratch experiment: Transfer cells from the logarithmic growth phase and introduce them into a six-well plate. Once the cell density reaches 90%, use a sterile 10 μL pipette to create a perpendicular scratch in each well, crossing the horizontal line made by the marker pen. Continue to incubate the cells in a 37 °C, 5% CO2 environment, and then at the designated time, remove the cells and capture photographs. In the Transwell experiment, the cells were deprived of food for a whole night. Then, the concentration of the cells was modified, and the Transwell chamber was immersed in a culture medium that included 10% fetal bovine serum. Finally, the cell suspension was introduced into the top chamber of the Transwell chamber. Following a period of 36–48 hours, the chamber underwent repairs, was subjected to staining, photographed using a microscope, and the documentation process was completed.

### Statistical analysis

The Kaplan-Meier survival curves were used to assess the disparities in survival rates between the two groups. The prognostic value was assessed using both univariate and multivariate Cox regression analysis. The Spearman correlation analysis was used to evaluate the correlation. R was used to conduct all statistical analyses.

## Results

### Unsupervised clustering of genes associated to regulated cell death

**Figure 1:**
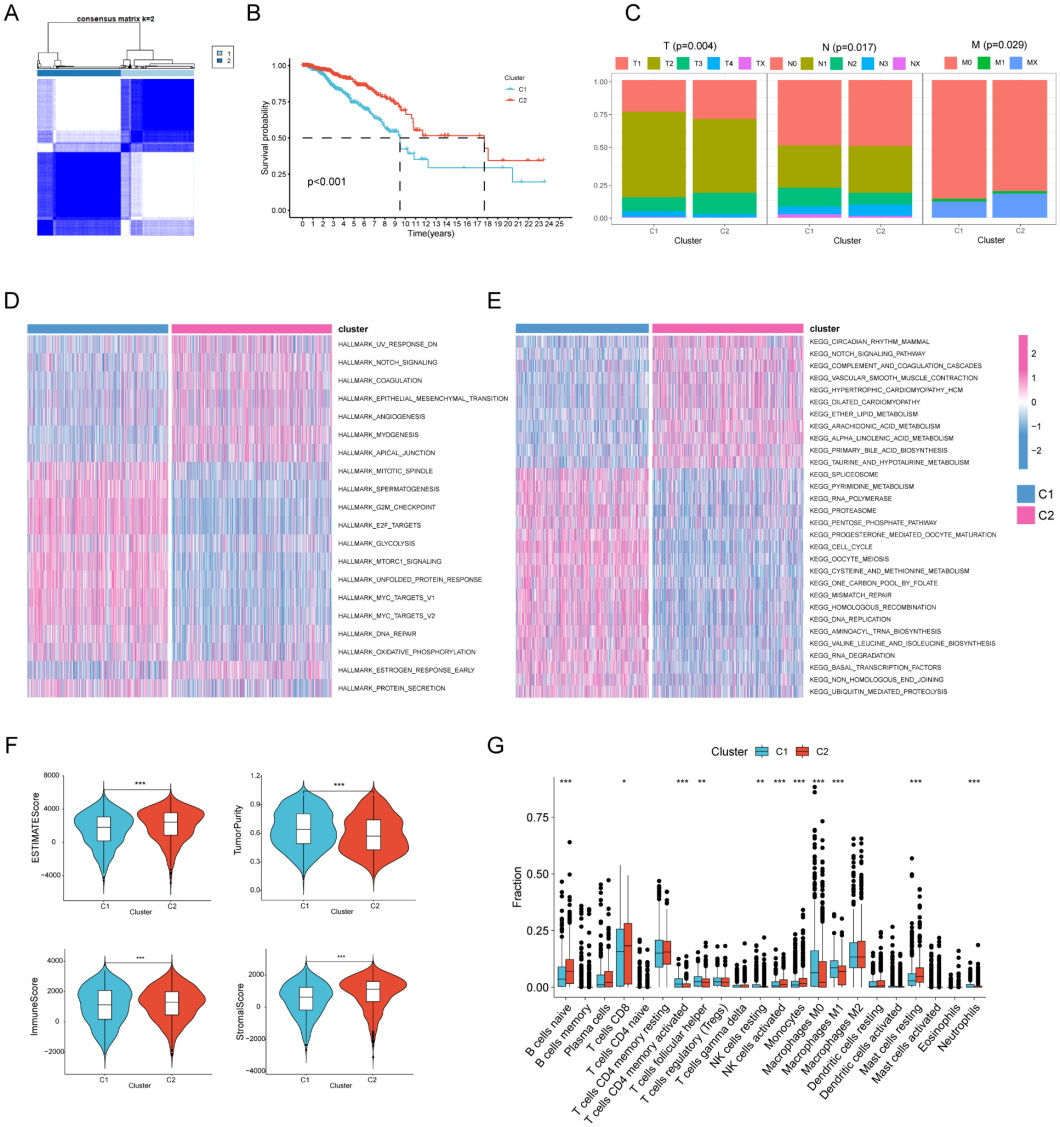
Unsupervised cluster analysis of regulated cell death genes in gastric cancer. **(A)** When k = 2, gastric cancer patients (n=375) are divided into two clusters based on regulated cell death-related genes (C1n=187, C2n=188). **(B)** Kaplan-Meier curves show the prognosis of gastric cancer patients in the two clusters (C1n=187, C2n=188). **(C)** The proportion of clinical and pathological characteristics between the two clusters (C1n=187, C2n=188). **(D, E)** Heat maps of HALLMARK pathways and KEGG pathways enriched between the two clusters through enrichment analysis. **(F)** Differences in tumor purity, ESTIMATEScore (Sum of stromal cell scores and immune cell scores ), immune score and stromal score between the two clusters (C1n=187, C2n=188). **(G)** The CIBERSORT algorithm was used to analyze differences in common immune cells between the two clusters (C1n=187, C2n=188).

A collection of 1628 genes associated with regulated cell death was gathered to investigate the subtypes of gastric cancer. The research revealed that when k = 2, the disparities between the subgroups were very significant, suggesting that gastric cancer patients (TCGA-STAD n= 375) may be effectively classified into two subgroups based on regulated cell death-related genes (**Figure 1A**). The survival curve demonstrated a notable disparity in the overall duration of survival between the two groupings. Patients with gastric cancer in Cluster C1 had a negative prognosis, but those in Cluster C2 had a positive prognosis (**Figure 1B**). Through a comparison of clinical and pathological features, namely the T, N, and M stages, we observed that C2 had lower T, N, and M stages compared to C1. This suggests that patients in C1 had a greater malignant phenotype (**Figure 1C**). Subsequently, we conducted gene set enrichment analysis to examine the enriched pathways between the two clusters. The analysis revealed that Cluster 1 exhibited enrichment in pathways such as CELL CYCLE, G2M CHECKPOINT, and OXIDATIVE PHOSPHORYLATION, whereas Cluster 2 showed enrichment in pathways such as NOTCH and ANGIOGENESIS (**Figures 1D, E**). Subsequently, we conducted an analysis of the immunological features shown by the two clusters. The tumor purity of C2 was markedly reduced compared to C1. This suggests that the amount of immune infiltration in C2 was greater than in C1 (**Figure 1F**). Ultimately, the CIBERSORT algorithm was used to assess the number of immune cells that had infiltrated. The findings indicated that the infiltration level of immune cells was greater in C2 (**Figure 1G**).

### Development of RCDRI in individuals with gastric cancer

Through the application of diverse machine learning techniques, including univariate Cox regression, lasso, and multivariate Cox regression analysis, we have successfully identified four genes (CD36, SERPINE1, TRIML2, and GRP) that are associated with regulated cell death (RCD). Additionally, we have developed the Regulated cell death index (RCDRI) and its derivatives, which are directly linked to RCD. The Regulated cell death index (RCDRI) for each patient was calculated by our model using the following formula: RCDRI = (0.0820*CD36 exp.) + (0.0247*SERPINE1 exp.) + (0.2835*TRIML2 exp.) + (0.0698*GRP exp.).

### Validation of the external data set and assessment of the clinical significance of RCDRI

**Figure 2:**
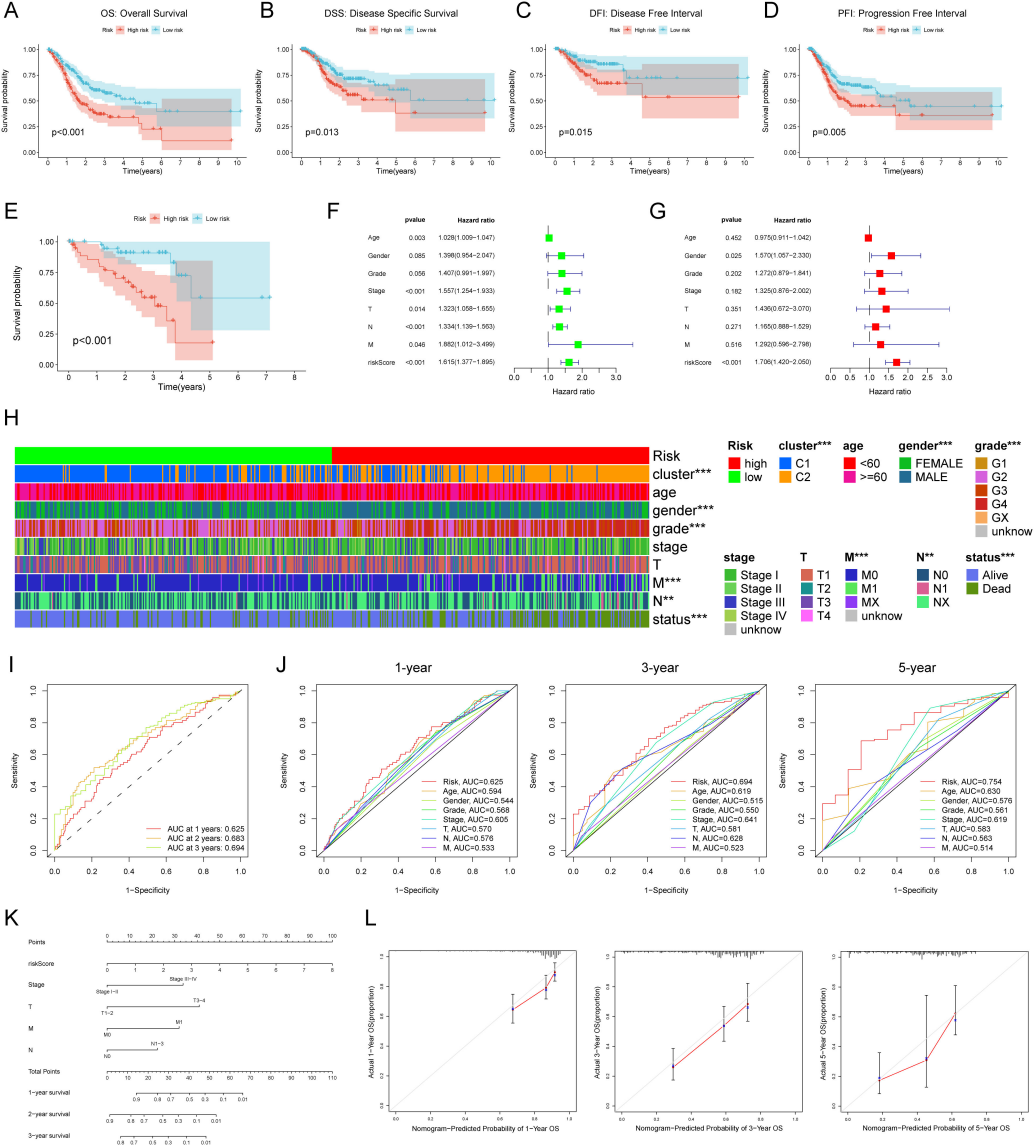
External data set validation and clinical relevance of Regulated Cell Death-Related Index (RCDRI). Kaplan-Meier survival curves for high/low RCDRI in TCGA data sets **(A–D)** and GEO external data sets **(E)**. Univariate **(F)** and multivariate **(G)** Cox regression analysis of RCDRI and other clinical traits. **(H)** Differences in RCDRI between common clinical characteristics. **(I)** ROC curves of RCDRI at 1 year, 3 years and 5 years. **(J)** The three ROC curves for RCDRI represent its comparison with other clinical characteristics at 1, 3 and 5 years, respectively. **(K)** Line plot predicting the prognosis of gastric cancer patients. **(L)** Calibration curves of the line plot for 1-, 3- and 5-year overall survival probability in the TCGA cohort.

Our analysis revealed a significant correlation between high RCDRI and unfavorable prognosis (p<0.05, **Figures 2A–D**) as shown by the Kaplan-Meier survival curves. These curves were generated using several statistical techniques to calculate patient survival time, including Overall Survival, Disease Specific Survival, Disease Free Interval, and Progression Free Interval. Afterwards, we used the GEO dataset (GSE84437) as a validation cohort. The Kaplan-Meier curve demonstrated that gastric cancer patients with high RCDRI had a significantly worse overall survival rate, comparable to the findings seen in the training group. Conversely, individuals with low RCDRI levels had a potentially favorable prognosis (**Figure 2E**). The results of both univariate and multivariate Cox regression analyses indicate that RCDRI may serve as an independent predictive factor in patients with gastric cancer (**Figures 2F, G**). We conducted an analysis to examine the variations in RCDRI across various clinical variables. Our findings indicate that the high/low RCDRI groups exhibit substantial differences in terms of distinct clusters, gender, grade, M, N, and survival status (**Figure 2H**). The findings indicated that RCDRI outperformed other clinical features in predicting the survival rate of gastric cancer patients, as shown by assessing the area under the curve values (**Figures 2I, J**). Ultimately, a nomogram was created to assess the prognosis of patients (**Figure 2K**). This indicates that the developed nomogram is very accurate in predicting patient prognosis (**Figure 2L**).

### RCDRI provides prognostic predictions for several types of cancer, including gastric cancer

**Figure 3:**
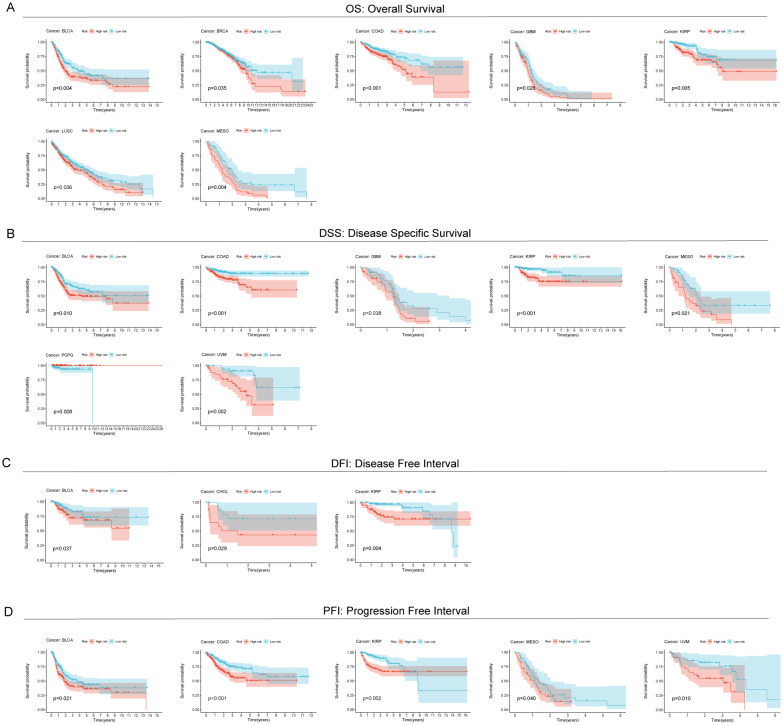
Predictive value of RCDRI in other cancers. Kaplan-Meier survival curves were used to compare the OS **(A)**, DSS **(B)**, DFI **(C)** and PFI **(D)** of patients with high/low RCDRI in pan-cancer.

In order to investigate the frequency of RCDRI in different forms of cancer, we used the aforementioned RCDRI model formula to determine the RCDRI values of patients with various cancer types across several cancers. We then graphed the Kaplan-Meier survival curves for the groups with high and low RCDRI values. Patients in the high RCDRI group showed a worse prognosis for Overall Survival (OS) in the following cancer types: BLCA, BRCA, COAD, GBM, KIRP, LUSC, and MESO (**Figure 3A**). In terms of DSS, patients belonging to the high RCDRI group exhibited worse DSS in the cases of BLCA, COAD, GBM, KIRP, MESO, and UVM. Conversely, individuals in the low RCDRI group showed worse DSS only in the case of PCPG (**Figure 3B**). Regarding the DFI, patients classified in the high RCDRI group had a more unfavorable Disease Free Interval in cases of BLCA, CHOL, and KIRP (**Figure 3C**). In terms of the PFI, patients in the high RCDRI group had a more unfavorable Progression Free Interval in the cases of BLCA, COAD, KIRP, MESO, and UVM (**Figure 3D**). The aforementioned findings demonstrate that RCDRI not only has a favorable impact in prognosticating the outcome of gastric cancer, but also possesses prognostic significance in other types of cancer.

### Tumor microenvironment in gastric cancer was conducted using RCDRI

**Figure 4:**
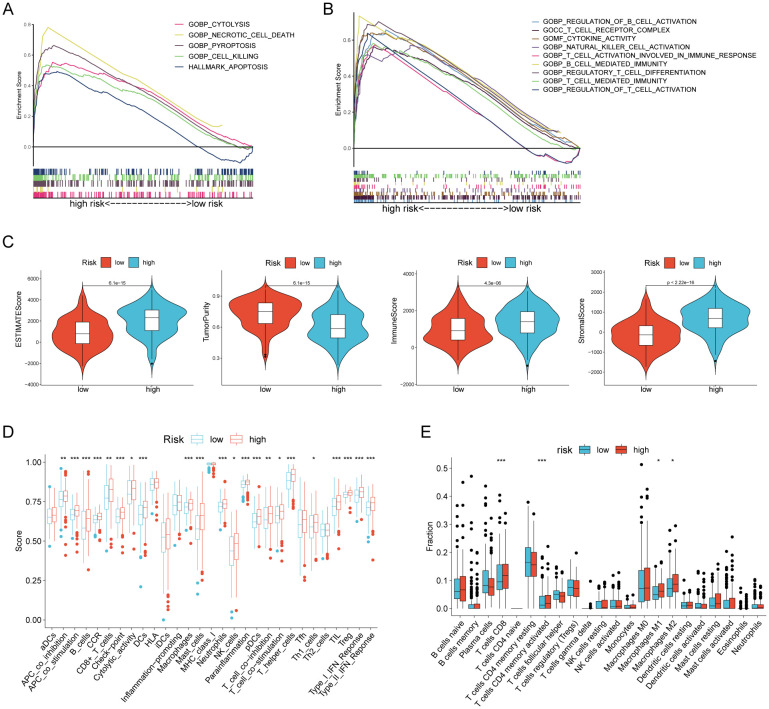
Evaluation of the tumor microenvironment based on the regulated cell death-related index. **(A, B)** GSEA analysis of patients in the high RCDRI group. **(C)** Differences in ESTIMATEScore, tumor purity, immune score, and stromal score between the high/low RCDRI groups. **(D)** ssGSEA algorithm to evaluate differences in immune cells and immune function between patients in the high/low RCDRI groups. **(E)** Immune cell differences between high/low RCDRI groups assessed by the CIBERSORT algorithm.

We then performed GSEA analysis on gastric cancer patients (TCGA-STAD n= 375)to discover the association between RCDRI and signaling pathways. The results of the GSEA analysis revealed that the high RCDRI group had a substantial enrichment in the signaling pathways related to Cytolysis, Necrotic cell death, Pyroptosis, Cell killing, and Apoptosis (**Figure 4A**). Furthermore, it was observed that tumors in the high RCDRI group exhibited a significant enrichment in immune function pathways, including the regulation of cytokine activity, natural killer cell activation, B cell mediated immunity, regulatory T cell differentiation, T cell mediated immunity, and regulation of T cell activation. This suggests a strong association between the high RCDRI group and the tumor immune microenvironment (**Figure 4B**).

We determined that the tumors in the high RCDRI group had decreased purity (**Figure 4C**). The ssGSEA algorithm revealed that the high RCDRI group exhibited superior immune cell infiltration and immune-related activities compared to the low RCDRI group. Specifically, the high RCDRI group had substantially elevated levels of immunological checkpoint compared to the low RCDRI group (**Figure 4D**). According to the CIBERSORT algorithm, the high RCDRI group exhibited considerably elevated numbers of immune-stimulating CD8 T cells compared to the low RCDRI group. This indicates that the high RCDRI group had strong immune infiltration features, as seen in **Figure 4E**.

### The effectiveness of RCDRI in predicting the effectiveness of immunotherapy

**Figure 5:**
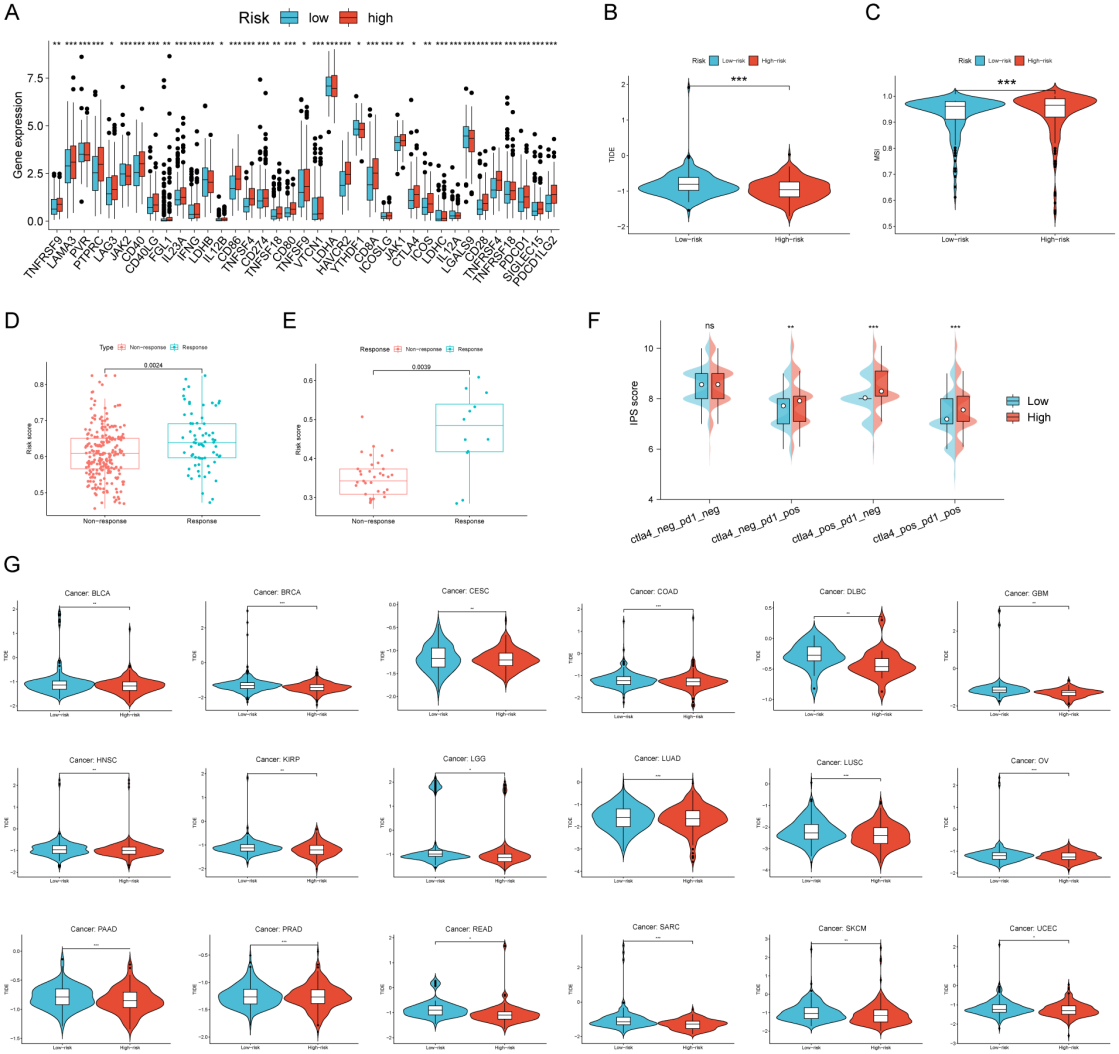
The efficacy of RCDRI in predicting immunotherapy efficacy. **(A)** Differences in common immune checkpoint expression levels between high/low RCDRI groups. **(B, C)** Differences in TIDE score and MSI score between high/low RCDRI groups. **(D, E)** Differences in RCDRI score between mmunotherapy response and non-response groups in the IMvigor 210 dataset and the Kim dataset. **(F)** Difference in IPS score between high/low RCDRI groups. **(G)** RCDRI score for evaluating immunotherapy efficacy in pan-cancer from TCGA databases. TIDE, Tumor immune dysfunction and exclusion.

In order to investigate the connection between RCDRI and the immunological milieu, we analyzed the variations in the expression levels of typical immune checkpoints between the high/low RCDRI groups. The findings indicated that the levels of immunological checkpoints were notably elevated in patients with high RCDRI compared to those with low RCDRI (**Figure 5A**). A lower TIDE score was correlated with an increased probability of responding positively to immunotherapy and experiencing extended longevity (18). Patients with high RCDRI had lower TIDE scores and greater MSI compared to patients with low RCDRI. This suggests that patients with high RCDRI may have more favorable responses to immune checkpoint blockade treatment than those with low RCDRI (**Figures 5B, C**). Subsequently, we used external immunotherapy datasets (IMvigor 210 dataset and Kim dataset) to predict immunotherapy response in high/low RCDRI groups. The results showed that the RCDRI scores of the immunotherapy response group were significantly higher than those of the immunotherapy non-response group. This suggests that high RCDRI scores may be closely associated with improved immunotherapy efficacy in gastric cancer patients. (**Figures 5D, E**). Subsequently, we investigated the correlation between the RCDRI group and the immunological phenotype score (IPS), which serves as a measure of patients’ response to anti-PD1 and/or anti-CTLA4 treatment. Our analysis revealed that the IPS score was significantly elevated in the high RCDRI group, suggesting that patients in this group may have a more favorable response to immunotherapy (**Figure 5F**). Ultimately, we assessed the predictive capability of the RCDRI score in determining the effectiveness of immunotherapy by assigning RCDRI scores to individuals with various types of cancer. The study found significant variations in TIDE scores between the low and high RCDRI score groups in multiple cancer types. The TIDE scores of the low and high RCDRI score groups differed significantly, suggesting that the RCDRI score can be used to assess the effectiveness of immunotherapy across various cancers. Furthermore, the high RCDRI group exhibited a more favorable response to immunotherapy (**Figure 5G**). These findings indicate that RCDRI may serve as a predictive tool for assessing the effectiveness of immunotherapy. Moreover, individuals in the high RCDRI group are more inclined to exhibit positive responses to immunotherapy.

### The effectiveness of RCDRI in predicting the sensitivity of drugs

**Figure 6:**
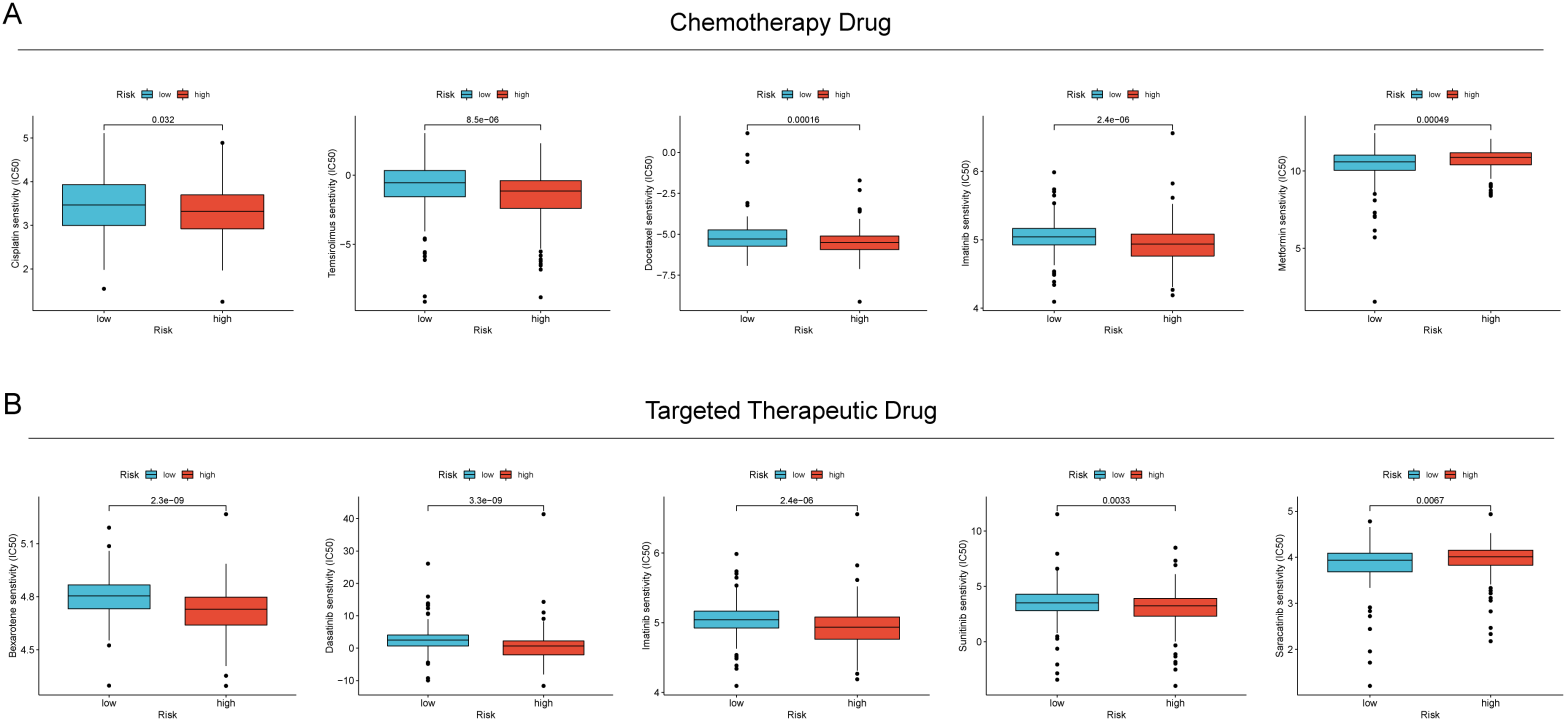
The value of RCDRI in predicting drug sensitivity. **(A)** The difference in IC50 values of common chemotherapeutic drugs between the high/low RCDRI groups. **(B)** The difference in IC50 values of common targeted therapeutic drugs between the high/low RCDRI groups.

The relationship between RCDRI and drug sensitivity was investigated by determining the IC50 values of commonly used chemotherapeutic medicines and targeted therapeutic medications, and comparing them with the RCDRI group. We observed significant disparities in the efficacy of several chemotherapeutic and targeted medications for gastric cancer when comparing the high and low RCDRI groups. For instance, the IC50 value of cisplatin was notably lower in the high RCDRI group compared to the low RCDRI group. This suggests that patients in the high RCDRI group may exhibit a more favorable response to cisplatin treatment. The IC50 value of Saracatinib in the low RCDRI group exhibited a considerably lower value compared to the high RCDRI group. This suggests that patients in the low RCDRI group shown a more favorable response to targeted treatment with Saracatinib. (See **Figures 6A, B**).

### Suppression of TRIML2 hampers the growth and movement of gastric cancer cell lines

**Figure 7:**
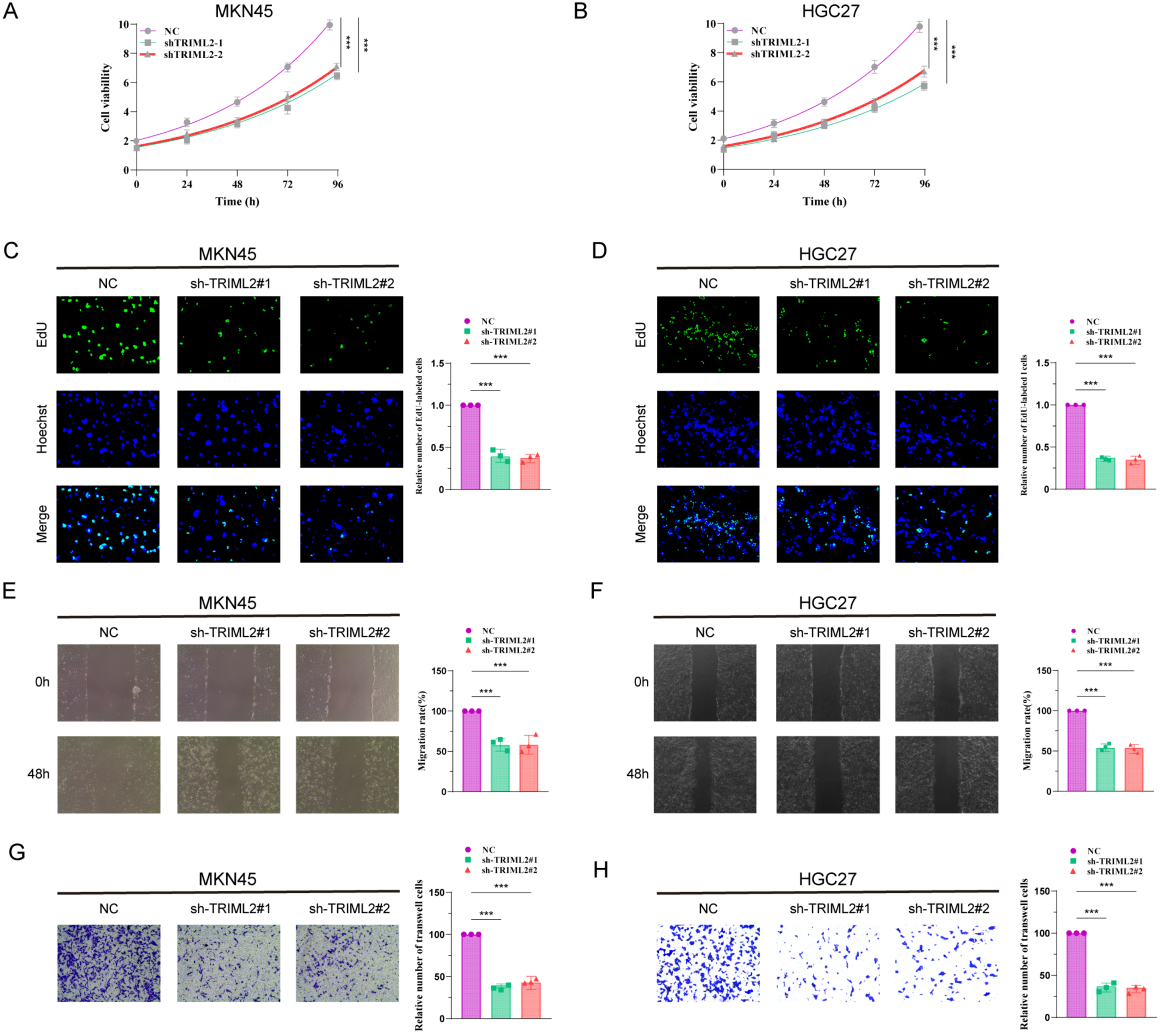
Knockdown of TRIML2 inhibits the proliferation and migration of gastric cancer cell lines. **(A, B)** CCK8 assay shows that the number of TRIML2-knockdown cells is reduced compared to sh-NC cells at 0h, 24h, 48h, 72h and 96h. **(C, D)** EdU assay shows that TRIML2-knockdown cells may have a weaker ability to form than NC cells. **(E–H)** Cell scratch experiments and Transwell experiments were used to observe changes in the migration and invasion abilities of TRIML2-knockdown cells compared to NC cells.

Initially, we created stable cell lines *in vitro* by suppressing the expression of TRIML2 in MKN45 and HGC27 gastric cancer cell lines. The cell count in the TRIML2-silenced group was consistently lower than that in the NC group at 0 h, 24 h, 48 h, 72 h, and 96 h in the CCK8 experiment. This suggests that the cells’ capacity to proliferate was diminished after TRIML2 was suppressed (**Figures 7A, B**). The TRIML2-silenced strain exhibited a reduction in clonogenic capacity relative to the sh-NC strain in the EdU experiment (**Figures 7C, D**). In both the cell scratch experiment and Transwell experiment, the strain with silenced TRIML2 exhibited a reduction in the quantity of cells that migrated and invaded, in comparison to the NC strain. This suggests a decline in the capacity to migrate and invade (**Figures 7E–H**).

## Discussion

Targeting regulated cell death in tumors is presently a crucial method in cancer therapy research. Potential pharmacological targets include many regulated cell death processes (19). Consequently, investigating the mechanisms and roles of different regulated cell death pathways might provide crucial groundwork for cancer therapy (20, 21). This work included the creation of a regulated Cell Death-Related Index (RCDRI) using the essential genes CD36, SERPINE1, TRIML2, and GRP, which are associated with the four different types of regulated cell death. Our findings indicate that RCDRI serves as a reliable marker for classifying gastric cancer. Additionally, it has a strong ability to accurately predict the prognosis and effectiveness of immunotherapy in gastric cancer patients. Ultimately, we confirmed via laboratory testing that suppressing TRIML2 hinders the growth and infiltration of stomach cancer cells.

Tumors have the capacity to modify their surrounding tumor microenvironment, enabling them to evade immune monitoring, which is directly linked to the formation and progression of tumors (22–24). This conclusion was reached through the utilization of the ESTIMATE algorithm, GSEA enrichment analysis, and ssGSEA algorithm. Subsequently, we conducted a comparison of the expression levels of immune checkpoints between the high and low RCDRI groups. Our findings revealed that patients in the high RCDRI group exhibited significantly elevated levels of immune checkpoints compared to those in the low RCDRI group. This observation suggests that tumor cells in the high RCDRI group may possess a highly active tumor immune microenvironment. We used the IPS score and TIDE score of anti-PD-1 and anti-CTLA-4 to forecast the effectiveness of immunotherapy in gastric cancer patients categorized as high or low RCDRI group. Our findings suggest that patients in the high RCDRI group may have superior immunotherapy efficacy compared to those in the low RCDRI group. Conversely, we assessed the correlation between RCDRI and drug sensitivity by analyzing the IC50 values of commonly used chemotherapeutic and targeted medicines. Our research revealed that RCDRI has the ability to differentiate the responsiveness of gastric cancer to widely used chemotherapeutic and targeted medications. The findings indicate that RCDRI has the potential to assess the effectiveness of immunotherapy and chemotherapy medications in individuals diagnosed with gastric cancer. This has significant implications for improving the treatment of gastric cancer patients in the future.

The Regulated Cell Death-Related Index (RCDRI) consists of four genes associated with regulated cell death: CD36, SERPINE1, TRIML2, and GRP. Recent studies have shown that fatty acids promote the metastatic potential of GC cells by upregulating CD36 through increased O-GlcNAcylation levels. The increase in O-GlcNAcylation levels promotes CD36 transcription by activating the NF-κB pathway and enhances its fatty acid uptake activity by directly modifying CD36 at positions S468 and T470 (25). Additionally, multiple studies have shown that CD36 can influence various pathological processes, such as ovarian cancer and fatty liver disease, through ferroptosis (26, 27). CD36 promotes iron accumulation and dysfunction in CD8+ T cells via the p38-CEBPB-TfR1 axis (28). Serpin Family E Member 1 (SERPINE1) is highly expressed in GC tissues and metastatic lesions. CEBPb activates SERPINE1 transcription through an autocrine mechanism, triggering the PI3K/AKT and EMT signaling pathways, thereby enhancing GC cells’ resistance to anoikis and metastatic potential. Additionally, SERPINE1 binds to lipoprotein receptor-related protein 1 (LRP1) via a paracrine mechanism, inhibiting CD8+ T cell infiltration and function in the tumor microenvironment (TME) and promoting M2 macrophage polarization (29). Wang et al. found that TRIML2 regulates the occurrence and progression of GC tumors (30). In addition, TRIML2 also enhances p53 SUMOylation and regulates the transactivation of pro-apoptotic genes (31).Research has shown that after Gastrin Releasing Peptide (GRP) binds to its receptor GRPR, GRPR can interact with Toll-like receptor 4 to activate STAT1, which binds to the promoters of MLKL and CCL2, inducing processes such as necrotic apoptosis, necrotic inflammation, and macrophage recruitment (32).The above literature indicates that the proteins encoded by the four genes that constitute the RCDRI index jointly influence gastric cancer progression and cell death pathways through different pathways. For example, CD36 mainly influences fatty acid metabolism through metabolic pathways, SERPINE1 influences the PI3K/AKT and EMT signaling pathways, TRIML2 is crucial for p53-mediated apoptosis, and GRP binds to its receptor to activate downstream pathways.

In our study, the high RCDRI group exhibited higher tumor invasiveness and greater immune infiltration activity. However, some current studies suggest that metastatic tumors may not respond effectively to immunotherapy (33). Therefore, it is necessary to explain why invasive tumors demonstrate better responses to immunotherapy, which may be attributed to the following factors: (1) Tumor cell characteristics: Invasive tumor cells typically exhibit higher mutation rates, leading to the production of more tumor-specific antigens. These antigens can be recognized by the immune system as “non-self” components, thereby activating immune cells to more effectively identify and attack tumor cells. Additionally, invasive tumor cells may undergo phenotypic and functional changes during growth and metastasis, enhancing their immunogenicity (34–38). (2) Tumor microenvironment: Aggressive tumors often lead to increased infiltration of immune cells in the tumor microenvironment, and as aggressive tumors develop, they may gradually disrupt immune-suppressive factors in the tumor microenvironment (39–41). (3) Mechanism of action of immunotherapy: For aggressive tumors, due to their stronger immunogenicity, immune checkpoint inhibitors can more effectively activate immune cells to kill tumor cells (42–45).

This study has certain limitations. First, our analysis data was obtained from public databases, which may lead to case selection bias. In addition, it is still necessary to collect a large amount of clinical case data for evaluation to further verify the accuracy of our research results. Finally, further *in vivo* and *in vitro* experiments are needed to verify the functional roles of the three genes (CD36, SERPINE1, and GRP) in gastric cancer that were used to construct the RCDRI index.

## Conclusion

To summarize, our thorough examination of gastric cancer, using the RCDRI derived from genes associated with regulated cell death, has shown that the RCDRI is a reliable tool for predicting the prognosis and effectiveness of immunotherapy in gastric cancer patients. This conclusion has been confirmed by external datasets. From the standpoint of regulated cell death, we have identified novel prognostic and therapeutic biomarkers as well as targeted small molecule medications for gastric cancer. These findings provide valuable insights for the future development of precise treatments for gastric cancer. During a time when immunotherapy shows significant potential for treating cancer, RCDRI offers direction for the clinical identification and personalized complete therapy of gastric cancer.

## Data Availability

The original contributions presented in the study are included in the article/**Supplementary Material**. Further inquiries can be directed to the corresponding author.

## Supplementary material

Supplementary Table 1 RCD-related genes.

Supplementary Table 2 General characteristics of the included studies.

Supplementary Table 3 The primer sequences used for qRT-PCR and shRNA sequences.
